# The Microbiome in the Obesity-Breast Cancer Axis: Diagnostic and Therapeutic Potential

**DOI:** 10.3390/pathogens12121402

**Published:** 2023-11-29

**Authors:** Dimiter Avtanski, Varun Reddy, Radoslav Stojchevski, Nikola Hadzi-Petrushev, Mitko Mladenov

**Affiliations:** 1 Friedman Diabetes Institute, Lenox Hill Hospital, Northwell Health, New York, NY 10022, USA; rstojchevski@northwell.edu; 2 Feinstein Institutes for Medical Research, Manhasset, NY 11030, USA; 3 Donald and Barbara Zucker School of Medicine at Hofstra/Northwell, Hempstead, NY 11549, USA; 4 New York Institute of Technology, College of Osteopathic Medicine, Old Westbury, NY 11545, USA; vreddy04@nyit.edu; 5 Faculty of Natural Sciences and Mathematics, Institute of Biology, Ss. Cyril and Methodius University, 1000 Skopje, North Macedonia; nikola@pmf.ukim.mk (N.H.-P.); mitkom@pmf.ukim.mk (M.M.)

**Keywords:** microbiome, obesity, breast cancer, dysbiosis, diagnostic, therapeutic, diet, antibiotics, breast, inflammation

## Abstract

A growing body of evidence has demonstrated a relationship between the microbiome, adiposity, and cancer development. The microbiome is emerging as an important factor in metabolic disease and cancer pathogenesis. This review aimed to highlight the role of the microbiome in obesity and its association with cancer, with a particular focus on breast cancer. This review discusses how microbiota dysbiosis may contribute to obesity and obesity-related diseases, which are linked to breast cancer. It also explores the potential of the gut microbiome to influence systemic immunity, leading to carcinogenesis via the modulation of immune function. This review underscores the potential use of the microbiome profile as a diagnostic tool and treatment target, with strategies including probiotics, fecal microbiota transplantation, and dietary interventions. However, this emphasizes the need for more research to fully understand the complex relationship between the microbiome, metabolic disorders, and breast cancer. Future studies should focus on elucidating the mechanisms underlying the impact of the microbiome on breast cancer and exploring the potential of the microbiota profile as a biomarker and treatment target.

## 1. Introduction

A large body of evidence has demonstrated a significant link between obesity and cancer risk. Adipose tissue, conventionally viewed as a passive reservoir for energy storage, is now recognized as a highly secretory endocrine organ that produces various pro- and anti-inflammatory cytokines, estrogens, and other bioactive molecules [1,2]. Obesity, characterized by adipose tissue hypertrophy (increase in adipocyte size) and hyperplasia (increase in adipocyte number), causes the dysregulation of adipose tissue hormonal production, leading to chronic low-grade inflammation that can contribute to the initiation and progression of breast cancer, particularly among postmenopausal women [3,4,5,6,7,8,9]. Furthermore, obesity-related metabolic changes can influence the composition of the gut microbiome, leading to dysbiosis, which may further affect breast cancer risk and outcomes.

Over the past two decades, following advancements in DNA sequencing technologies, the microbiome has been recognized as a major factor in maintaining health. The interaction between the microbiome and the host organism is a dynamic bidirectional relationship, where disruptions in the microbiome reflect the host’s health and vice versa: modifications to the health status of the host lead to corresponding microbiome changes.

Breast cancer is the most common type of cancer in women worldwide, increasingly affecting the younger population. While patients with breast cancer are currently experiencing higher survival and decreasing recurrence rates, the mortality remains high [10]. Despite well-known risk factors for breast cancer development, such as genetic predisposition, sex, age, estrogen levels, and obesity, the cause of more than half of new cases remains unknown [11]. Ample evidence suggests that the microbiome may play a significant role, among multiple factors. As most studies have concentrated on the gut microbiome, little is known about the influence of other microbial communities residing throughout the body. The microbiome in the breast is characterized by a different composition, and it is not incorrect to speculate that it plays various roles. 

This review aims to present a brief, up-to-date overview of the role of the microbiome in breast cancer pathogenesis and explore how factors influencing its composition may impact disease development and progression, thus providing an evolutionary perspective on breast cancer.

## 2. Characteristics of the Breast Microbiome

Our bodies are complex entities composed of trillions of individual cells, including entire communities of symbiotic microorganisms (bacteria, archaea, and eukarya) that collectively constitute our microbiome [12]. The microbiome is now recognized as a pivotal determinant of an individual’s health, as its disturbance has been linked to various diseases [10,13,14,15,16].

The breast microbiome is a unique niche characterized by distinct microbial communities, composition, and characteristics [17,18]. It is believed to be formed predominantly from the migration of bacteria from the areola, as well as through the entero-mammary pathway, via immune cells translocating gut bacteria to secondary lymph nodes and progressing to the breast tissue through the lymphatic circulation or the blood [19,20,21]. The breast microbiome is shaped by the maternal diet, most prominently by dietary soluble fibers and plant and animal proteins [22]. Extensive studies of the breast milk microbiota have revealed a vast diversity of microbes, such as *Staphylococcus*, *Streptococcus*, *Lactobacillus*, *Pseudomonas*, *Bifidobacterium*, *Corynebacterium*, *Enterococcus*, *Acinetobacter*, *Rothia*, *Cutibacterium*, *Veillonella*, and *Bacteroides* [23]. The breast microbiome varies among races and ethnicities, as reported by Smith et al. [24] and others.

Although the role of the microbiome in breast cancer pathogenesis is gaining increasing attention, it is critical to recognize the inherent obstacles associated with microbiome research in this context [25]. These obstacles include, but are not limited to, variations in sample collection methods, differences in DNA extraction protocols, potential contamination issues, and the need for robust bioinformatics and statistical analyses to interpret complex datasets [26]. All these factors affect the replicability of published findings and need to be addressed adequately to guarantee the consistency and comparability of the obtained results. Functional testing of the statistical correlations is necessary to validate these findings [18,20]. 

## 3. Role of the Microbiome in Obesity-Induced Inflammation

Obesity is currently a pandemic affecting 650 million individuals worldwide, in addition to almost two billion categorized as overweight [27]. Predictions project that overweight and obesity will continue to increase in the foreseeable future.

The consistent energy overload mainly affects visceral white adipose tissue. Adipose tissue hypertrophy impairs normal adipocyte differentiation and secretion and stimulates tissue infiltration of immune cells, resulting in elevated proinflammatory cytokine secretion and chronic low-grade inflammation [28], leading to the development of metabolic conditions such as metabolic syndrome, dyslipidemia, insulin resistance, and type 2 diabetes [29,30,31]. The level of adiposity also strongly correlates with an increased incidence and worse outcomes in many different types of cancer [32]. Obesity is thought to be related to a 30% increase in breast cancer risk [33,34], and various studies have found intriguing associations between microbiota and obesity [35]. Although obesity plays a protective role in the development of breast cancer in premenopausal (particularly European) women, it shows a strong positive correlation with breast cancer risk in postmenopausal settings [36]. Among the multiple factors involved in this association, cytokines released by the white adipocytes per se, or activated macrophages, may directly promote the invasive potential and aggressiveness of breast cancer cells [37,38].

The gut microbiome composition is tightly modulated by metabolic signals and plays a significant role in the development of obesity. The level of adiposity is positively associated with changes in the microbiome composition (referred to as dysbiosis), characterized by generally reduced diversity and a shift in the abundance of dominant species [39,40,41,42]. For example, a cohort study involving primary school students in China revealed that obese children had lower species diversity and a relative abundance of typically dominant bacterial strands but a higher abundance of other genera [43]. Obese leptin-deficient (*ob*/*ob*) mice have a higher *Firmicutes*/*Bacteroidetes* (F/B) bacterial ratio than their wild-type counterparts [44]. Similar changes in the F/B ratio were observed in obese and lean humans [39,44,45]. Differences in abundance between lean and obese individuals have also been detected in other bacterial groups, such as those from the *Oscillospira* genus or the *Christensenellaceae* family [46,47]. Lv et al. [42] demonstrated a linear relationship between the body mass index (BMI) and several bacterial families (*Porphyromonadaceae*, *Acidaminococcaceae*, *Rikenellaceae*, and *Desulfovibrionaceae*). White et al. [48] suggested that gut microbiota is a modifiable factor linked to early rapid weight gain during infancy, and early weight gain has been identified as a risk factor for obesity during adulthood. The connection between the gut microbiome and adiposity extends to preterm infants, where the microbial composition was found to correlate with weight gain and subsequent growth, showing the influence of the microbiota from the earliest stages of life [49]. Similar to the gut microbiome, the breast tissue microbiome shows disparities between lean and obese individuals, with obese individuals exhibiting reduced bacterial diversity [17,18].

Dietary patterns may cause gut dysbiosis, which can lead to chronic inflammation [12]. A growing body of evidence has revealed that obesity-induced inflammation is associated with changes in microbiome composition. For example, using a high-fat diet (HFD)-induced obesity C57Bl/6 mouse model, Albornoz et al. [50] showed that obesity increases the susceptibility, pulmonary inflammation, and interferon-gamma (INF-γ) levels, following an infection with *Mycobacterium tuberculosis*. Gut bacteria metabolize dietary fiber into short-chain fatty acids (SCFAs), primarily butyrate, acetate, and propionate [51]. Butyrate has beneficial effects against obesity, including the promotion of lipolysis and an increase in energy expenditure. It also possesses anti-inflammatory properties by inhibiting proinflammatory cytokine production and reducing the translocation of lipopolysaccharides (LPSs) from the gut lumen to the bloodstream [52,53]. Butyrate also inhibits the expression of nitric oxide synthase (NOS) in intestinal cells by activating peroxisome proliferator-activated receptor gamma (PPARγ) signaling, thus limiting the growth of certain bacteria (such as those of the *Enterobacteriaceae* family) [54].

## 4. Microbiota and Breast Cancer

The microbiome profile has been linked to many types of cancers (stomach, colon, liver, lung, and skin, among others). The most robust connections are observed in cancers of the gastrointestinal tract, which are primarily associated with *Helicobacter pylori* and *Fusobacterium* bacteria [18,55,56,57,58,59,60].

**Figure 1:**
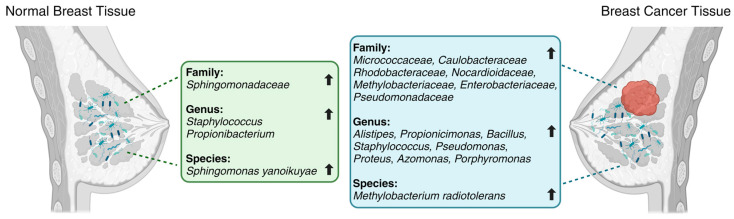
Differences in microbial taxa between normal breast tissue (on the **left**) and breast cancer tissue (on the **right**) per various studies. (Created with BioRender).

Breast cancer patients are characterized by decreased microbial diversity, as reported in several studies [19,61,62,63]. Early observational studies detected impaired intestinal microbiota in breast cancer patients, represented by a higher proportion of fecal *Enterobacteriaceae*, aerobic *Streptococci*, *Lactobacilli*, and anaerobic species such as *Clostridia*, *Lactobacilli*, and *Bacteroides* [64]. A comparative analysis by Xuan et al. [19] showed the enrichment of *Methylobacterium radiotolerans* in breast tumor tissues and *Sphingomonas yanoikuyae* in paired normal breast tissues (Figure 1). Using 16S rRNA gene amplicon sequencing, Chan et al. [65] investigated microbiota from nipple aspirates from healthy women and those with breast cancer and reported a higher incidence of *Sphingomonadaceae* in the healthy subject group and a higher proportion of the genus *Alistipes* in breast cancer patients (Figure 1). The microbiota of breast tissue adjacent to the tumor showed higher levels of the phylum *Bacteroidetes* and the genera *Bacillus* and *Staphylococcus* than those in healthy tissues [20]. Similarly, Meng et al. [66] analyzed breast tissue samples using needle biopsies from patients with breast cancer and benign tumors and observed an increase in the genus *Propionicimonas* and the families *Micrococcaceae*, *Caulobacteraceae*, *Rhodobacteraceae*, *Nocardioidaceae*, and *Methylobacteriaceae* (Figure 1). The microbial characterization of samples from 25 breast cancer patients showed lower levels of *Firmicutes* and *Bacteroidetes* and higher levels of *Proteobacteria*, *Actinobacteria*, and *Verrucomicrobia*, accompanied by a reduction in *Faecalibacterium prausnitzii* [67]. Another comparative study between patients with breast cancer and healthy individuals [11] demonstrated greater levels of *Enterobacteriaceae* and *Pseudomonadaceae* families (such as the genera *Pseudomonas*, *Proteus*, *Azomonas*, and *Porphyromonas*) in breast tumors and predominance of the genera *Staphylococcus* and *Propionibacterium* in healthy controls (Figure 1). A comparison of the breast tissues adjacent to the tumor showed a higher abundance of *Bacteroidetes* (*Bacillus* and *Staphylococcus*). Additionally, the F/B ratio was found to be significantly higher in patients with breast cancer than in controls [67].

Furthermore, microbial profiles vary during the progression of breast cancer. A comparison of the microbiome profiles of malignant tissues of different histological grades revealed that the development of breast cancer was associated with a decreased proportion of bacteria from the *Bacteroidaceae* family and an increased proportion of bacteria from the *Agrococcus* genus [66]. Stage I breast cancers exhibit an abundance of *Proteobacteria*, *Ruminococcaceae*, and *Hyphomicrobium*; stage II breast cancers show higher levels of *Euryarchaeota*, *Firmicutes*, *Spirochaetes*, and *Sporosarcina*, whereas stages III and IV breast cancers have high levels of *Thermi*, *Gemmatimonadetes*, *Tenericutes*, and *Bosea* [24].

Evidence suggests that shifts in microbial assemblages in the breast are related to breast cancer development, aggressiveness, and progression [18]. Using next-generation sequencing techniques and quantitative PCR analysis, Xuan et al. [19] demonstrated that breast tumor tissue has a reduced expression of antibacterial response genes, compared with adjacent healthy breast tissue. The observed dysbiosis in breast cancer suggests that bacteria may play a role in maintaining the normal cellular processes in the breast. Thus, it is speculated that the microbial components present in the breast may influence the local microenvironment. It is hypothesized that chronic exposure to low-residue antimicrobial drugs ingested from the diet could disrupt the gut microbiota equilibrium, which can contribute to corresponding physiological changes [68]. Dysbiosis caused by antibiotic use may increase the risk of breast cancer; however, more extensive studies are needed to confirm this hypothesis [69,70].

## 5. Etiology of Microbiome Dysbiosis in Breast Cancer

There are various mechanisms by which microbiota can influence breast cancer initiation and development. These include changes in adiposity, systemic estrogen levels, insulin resistance, dyslipidemia, and inflammation [71]. 

Adipose status is a major factor that affects microbial communities. Low gut microbial diversity occurs with obesity, insulin resistance, dyslipidemia, leukocytosis, and elevated levels of C-reactive protein (CRP), which are linked to breast cancer [71]. Hossain et al. [72] demonstrated that obesity is associated with increased incidence and worse prognosis in triple-negative breast cancer (TNBC) through various potential mechanisms, including the modulation of the gut microbiome. Using 16S rRNA sequencing and metagenomic analyses, the authors showed that obesity in TNBC decreases alpha diversity in the gut microbiome and is strongly correlated with functional profiles [72]. TNBC has been found to have the least taxonomic diversity among all breast cancer types, indicating a potential link between TNBC and the breast microbiome [73]. Levels of sex hormones have been shown to regulate the diversity of the gut microbiome [74]. Moreover, antimicrobial exposure during curative-intent treatment of TNBC has been linked to gut microbiome dysbiosis and decreased survival, suggesting a possible relationship between hormone levels and gut microbiome in TNBC [75]. Increased levels of hormones such as estrogens, insulin, insulin-like growth factor (IGF), and leptin have been associated with an increased cancer risk in obese individuals [76]. Studies have shown that the breast microbiome biomass decreases in breast cancer patients [77]. Additionally, distinct microbial communities have been observed in breast tissues of non-Hispanic Black and non-Hispanic White women, with differences in microbiome composition by race, breast cancer stage, or breast tumor subtype [24,78]. Dysbiosis of microbiota may contribute to obesity and obesity-related diseases through various mechanisms, including energy harvesting, direct effects on gene expression, and direct or indirect effects of chronic inflammation [79]. Dysbiosis of obesity is associated with hormonal changes. For example, estrogens (a risk factor for breast cancer) are elevated in obese individuals and modulated by microbiota [80]. Therefore, concomitant dysbiosis and obesity may increase the risk of breast cancer [81]. The enterohepatic recycling of estrogens by gut bacteria with increased β-glucuronidase (β-GUS) or β-glucosidase (β-Gluc) activity (*Firmicutes* (*Clostridium coccoides*, *Clostridium leptum*), *Actinobacteria* (*Bifidobacterium* sp.), or *Bacteroidetes* (*Bacteroides* sp.)) causes deconjugation of estrogen, which leads to increased levels of estrogens in circulation and an increased risk of breast cancer [82,83]. Several studies suggested that β-GUS may have a role in breast cancer development and progression [82,84,85,86]. The increase in β-GUS and β-Gluc activities in feces was found to be highly associated with bacteria from the *Clostridia* and *Ruminococcaceae* families [87].

The difference in the breast microbiota profile between lean and obese individuals suggests that the microbiome may contribute to chronic low-grade inflammation of adipose tissue, thus affecting the development, progression, and outcome of breast cancer [88]. The composition of the breast microbiota is influenced by multilayered interactions with the immune system [62]. The difference in breast microbiota between breast cancer subtypes and disease severity suggests a potential role for immunosuppression and tumor evasion by the immune system.

Gut bacteria influence systemic immunity [89,90,91]. In mice, targeted orogastric infection with *Helicobacter hepaticus* causes mammary neoplasia with an increased frequency [92,93]. The diversity of the gastrointestinal microbiome is closely associated with lymphocyte infiltration. Shi et al. [94] compared the gastrointestinal microbiome with the number of tumor-infiltrating lymphocytes (TILs) and found that breast cancer patients with higher gut microbial diversity showed an increased number of TILs. Disruption of the gut microbiota may lead to carcinogenesis via the modulation of immune function [95]. Animal model studies have shown that changes in immune function led to the initiation and progression of breast tumors. Mouse models with antibiotic-induced dysbiosis showed an increase in fibrosis and collagen deposition and induced higher myeloid cell infiltration into tumors, as well as normal adjacent breast tissue, at both the early and late stages of breast tumor progression [96]. The microbiome also affects tumor necrosis factor-alpha (TNFα)-mediated innate immune inflammatory responses, CD25+ regulatory T cells, and neutrophils [92,93]. Mice with advanced tumors showed elevated expression of CCL2 (*s.* MCP-1), IL-23, IL-6, and arginase-1 (ARG1). An unbalanced host immune response to enteric bacteria may promote the development of cancer within the gastrointestinal tract and epithelial cells distant from the gut [92,97,98,99,100,101,102,103].

Gut microbiome influences the development and progression of breast cancer. Perturbations in the gut microbiota (for example, due to antibiotic use) can provoke the elevation of free estrogen levels, thereby increasing the risk of breast cancer initiation [34]. The gut microbiome also affects the response to breast cancer treatment, including hormone therapy and chemotherapy [104].

Increased levels of bile acids have been observed in breast tumors and have been found to positively impact cancer survival by inhibiting tumor growth [105]. The intestinal microbiota converts bile acids from primary to secondary via deconjugation and 7α-dehydroxylation. Certain bile acids act like hormones by exerting their action in distant tissues by activating specific receptors such as the vitamin D receptor (VDR), Farnesoid X receptor (FXR), pregnane X receptor (PXR), and Takeda G-protein coupled receptor-5 (TGR5) [106]. For example, lithocholic acid, in particular, was found to decrease cancer cell proliferation due to its immune effects, partly through the activation of TGR5 [107]. Triggered by bile acids, FXR mediates breast cancer cell apoptosis and reduces aromatase expression, which is a local source of pro-proliferative estrogens [108].

## 6. The Microbiome as a Biomarker and Treatment Target

Based on current knowledge, the microbiome has emerged as a promising biomarker for evaluating breast cancer risk and prognosis or predicting the surgical outcomes and survival of patients with breast cancer [62,109,110,111]. For example, the F/B ratio can be used as an indicator of breast cancer risk [20,85]. Evaluation of the microbiome profile could have broad implications for the diagnosis and staging of breast cancer [66]. Meng et al. [66] showed that glycerophospholipid levels and ribosome biogenesis are higher in grade III breast cancers than in grades I and II. Additionally, the microbiome involved in human estrogen metabolism (also known as the *estrobolome*) can be used as another target for breast cancer treatment [85]. Microbial communities can alter the response to breast cancer therapy [112]. Gut microbe dysbiosis undermines the outcome of both immune and non-immune chemotherapeutic cancer treatment modalities [93,113,114]. The microbiota may potentially be targeted to enhance the efficacy and reduce the toxicity of conventional anticancer therapies. Taken together, the complex scenario linking microbiome composition to oncogenesis and the response to anticancer treatments defines the frame of a new “oncobiotic” perspective.

Probiotics have been shown to improve gut microbiota composition and function, suggesting their potential implications in cancer prevention and treatment [115]. *Lactobacillus* bacteria can modulate dysregulated SCFA levels in obesity by influencing other gut microbiota, energy absorption, and chronic low-grade inflammation [116]. Lactic acid bacteria (LAB) have been reported to exert anti-obesity effects. Thus, targeting the microbiome could be considered a potential treatment option for obesity [79]. Animal and cell-based studies have shown that probiotics may have anticancer effects because they can modulate the immune system and reduce obesity-induced low-grade chronic inflammation, potentially inhibiting cancer cell growth [115,117]. A study investigating the effect of oral administration of probiotics for 12 weeks, involving 18 patients with breast cancer, demonstrated an improved microbiome profile and serum tests (ANC (absolute neutrophil count), fasting blood glucose (FBG), and low-density lipoprotein cholesterol (LDL-C) levels) [118]. The most prominent changes observed in this study were for *Ruminococcus* and *Streptococcus* spp. The effects of probiotics, prebiotics, and synbiotics on breast cancer have been reviewed in randomized controlled trials [115,119]. A systematic review and meta-analysis of randomized clinical trials of probiotic and prebiotic use in breast cancer patients and survivors by Thu et al. [120] demonstrated the beneficial effects of a combination of pro- and prebiotics on obesity and dyslipidemia, as well as the reduction of TNFα levels, thus highlighting their potential against breast cancer. However, using probiotics to improve the gut microbiome as a treatment strategy for obesity is likely more complicated than anticipated and may require a long-term complex program [116].

Fecal microbiota transplantation (FMT) is another promising strategy for reducing obesity. Dietary interventions or FMT have emerged as promising strategies to help patients maintain a healthy weight [121]. FMT has been shown to reverse the effects of antibiotics and re-establish microbiota balance, resulting in the restoration of the normal functioning microbiome [122].

Furthermore, diet is known to influence the microbiota. The Mediterranean diet (characterized by a high content of plant-based foods and healthy fats) has been associated with a distinctive shift in the mammary gland microbiota, suggesting possible anti-breast cancer effects [123]. Long-term breast cancer risk is associated with diet-related plasma metabolic signatures involving exogenous steroid metabolites and microbiota-related compounds [124]. SCFAs are produced by two major groups of bacteria: *Firmicutes* bacteria produce butyrate, and *Bacteroidetes* bacteria produce acetate and propionate. It has been shown that SCFAs, more specifically butyrate, inhibit tumor growth [125]. A typical Western diet decreases the generation of SCFAs, causing a leaky gut and leading to an increase in inflammatory marker levels in the bloodstream, which results in the progression of breast cancer. Conversely, healthy diets with a higher fiber content may decrease inflammation by increasing SCFA production [126].

## 7. Conclusions

The microbiome comprises a significant proportion of multicellular organisms and is currently recognized as an essential contributor to the pathogenesis of metabolic diseases and cancer. The mechanisms by which microbiota influence breast cancer initiation and development are complex. Dysbiosis of the microbiome may contribute to obesity and obesity-related diseases associated with breast cancer. Furthermore, the microbiome can influence systemic immunity, potentially leading to carcinogenesis via the modulation of immune function. Patients with breast cancer often exhibit decreased microbial diversity, and accumulating evidence suggests that shifts in microbial assemblages are related to the development, aggressiveness, and progression of the disease.

The microbiome profile varies during breast cancer progression, indicating its potential as a biomarker for diagnosis and staging. This microbiome may also offer novel treatment options for patients with breast cancer. Treatment strategies include probiotics, FMT, and dietary intervention. Probiotics have shown promise in improving the composition and function of gut microbiota, which may result in the inhibition of cancer cell growth. Nutritional interventions, on the other hand, can influence microbiota, potentially decreasing inflammation and inhibiting tumor growth.

However, despite these promising findings, further research is needed to fully understand the complex relationship between the microbiome, metabolic disorders, and breast cancer [126]. This requires a multidisciplinary systems biology approach and evolutionary medicine thinking, combined with microbial ecology, immunology, cancer cell biology, and computational biology [127]. Future studies should focus on elucidating the mechanisms underlying the impact of the microbiome on breast cancer and exploring its potential as a biomarker and treatment target.

## Data Availability

No new data were created or analyzed in this study. Data sharing is not applicable to this article.

## Abbreviations

ANC	Absolute neutrophil count
ARG1	Arginase-1
β-Gluc	β-glucosidase
β-GUS	β-glucuronidase
CCL2	Chemokine (C-C motif) ligand 2 (*s*. Monocyte chemoattractant protein-1, MCP-1)
CRP	C-reactive protein
F/B	*Firmicutes/Bacteroidetes* bacterial ratio
FBG	Fasting blood glucose
FMT	Fecal microbiota transplantation
FXR	Farnesoid X receptor
HFD	High-fat diet
IGF	Insulin-like growth factor
IL	Interleukin
INF-γ	Interferon-gamma
LAB	Lactic acid bacteria
LDL-C	Low-density lipoprotein cholesterol
LPS	Lipopolysaccharide
NOS	Nitric oxide synthase
*ob/ob* mice	Leptin-deficient mice
PPARγ	Peroxisome proliferator-activated receptor gamma
PXR	Pregnane X receptor
SCFA	Short-chain fatty acid
TGR5	Takeda G-protein coupled receptor-5
TNBC	Triple-negative breast cancer
TNFα	Tumor necrosis factor-alpha
VDR	Vitamin D receptor
